# Sarcina ventriculi Bacteremia Complicating Aspiration Pneumonia: A Case Report

**DOI:** 10.7759/cureus.77676

**Published:** 2025-01-19

**Authors:** Shogo Saito, Yusuke Sasaki, Hiromi Nagashima, Tohru Fujiwara, Kiwamu Nakamura

**Affiliations:** 1 Department of Central Clinical Laboratory, Iwate Medical University Hospital, Yahaba, JPN; 2 Department of Critical Care and Disaster Medicine, School of Medicine, Iwate Medical University, Yahaba, JPN; 3 Division of Pulmonary Medicine, Department of Internal Medicine, School of Medicine, Iwate Medical University, Yahaba, JPN; 4 Department of Laboratory Medicine and Infectious Disease, School of Medicine, Iwate Medical University, Yahaba, JPN

**Keywords:** aspiration pneumonia, atrophic gastritis, bacteremia, chronic obstructive pulmonary disease, clostridium ventriculi, sarcina ventriculi

## Abstract

*Sarcina ventriculi*, a large anaerobic Gram-positive coccus that clusters in tetrads, is most commonly detected histologically in gastric biopsy specimens from patients with gastrointestinal disorders. Herein, we describe a rare case of bacteremia caused by *S. ventriculi* in an 89-year-old man. The patient had a history of cerebral infarction, atrophic gastritis, and chronic obstructive pulmonary disease and was receiving home oxygen therapy. He was admitted to our hospital with a right femoral neck fracture. Three days after femoral surgery, he developed aspiration pneumonia, and *S. ventriculi* was detected in the anaerobic blood culture bottle. A Gram-stained sputum smear showed large Gram-positive cocci (presumed to be *S. ventriculi*) clustered in tetrads. The patient was diagnosed with *S. ventriculi* bacteremia as a complication of aspiration pneumonia and recovered after ceftriaxone treatment. A literature review revealed only three previous case reports of *S. ventriculi* bacteremia. In previous case reports, the gastrointestinal tract was the presumed portal of entry into the blood. To our knowledge, *S. ventriculi* bacteremia has not previously been reported as a complication of lower respiratory tract infection.

## Introduction

*Sarcina ventriculi* is an anaerobic Gram-positive coccus belonging to the Clostridiaceae family [1]. *Sarcina *spp. are found as commensals in soil and human feces, especially in vegetarians [2]. *S. ventriculi* is most commonly detected in patients with gastroesophageal content stasis due to gastric outlet obstruction, gastroparesis, or delayed gastric emptying [3]. It is found primarily in the stomach, followed by the esophagus and duodenum. The severity of cases of infection varies, ranging from asymptomatic to life-threatening [4]. In severe cases, complications such as hemodynamic instability secondary to emphysematous gastritis and gastric perforation can occur [5]. It is primarily identified through histopathology of the gastrointestinal tract and is rarely detected using microbiological tests such as blood culture or sputum microscopy. The typical morphological features of *S. ventriculi* on Gram staining are useful for identification, but the identity must be confirmed using molecular techniques. Few cases of *S. ventriculi* bacteremia have been reported. Herein we report a unique case of *S. ventriculi* bacteremia that occurred as a complication of aspiration pneumonia and provide a brief literature review of *S. ventriculi* as a human pathogen.

## Case presentation

The patient was an 89-year-old man with a height of 160.0 cm and a weight of 51.8 kg, who had experienced a cerebral infarction 15 years previously. He had been taking cilostazol and mosapride citrate tablets for atrophic gastritis and receiving home oxygen therapy (2 L/min nasally) for chronic obstructive pulmonary disease (COPD). He visited the emergency room because of the acute onset of weakness in his right leg, which had started several hours previously. He was admitted to our hospital with a right femoral neck fracture. On admission, the patient’s vital signs were as follows: Glasgow Coma Scale: E4V5M6; blood pressure: 139/82 mmHg; heart rate: 74 beats/min; body temperature: 36.9°C; respiratory rate: 16 breaths/min; and peripheral oxygen saturation: 93% breathing 2 L/min oxygen via nasal prongs. Bipolar hip arthroplasty was performed on day 3 of hospitalization. On day 6, he became febrile and expectorated copious amounts of sputum. At that time, his body temperature was 39.6°C, his respiratory rate was 28 breaths/min, and his peripheral oxygen saturation had decreased to 80%, breathing 2 L/min oxygen via nasal prongs. His hematology and serum biochemistry test results are shown in Table 1.

**Table 1:** Blood test results at the time of developing aspiration pneumonia and bacteremia

Test (units)	Result	Reference range
White blood cells (× 10^9^/L)	7.3	3.3-8.6
Red blood cells (× 10^12^/L)	2.67	4.35-5.55
Hemoglobin (g/L)	86	137-168
Platelets (× 10^9^/L)	112	158-348
Total protein (g/L)	46	66-81
Albumin (g/L)	20	41-52
Urea nitrogen (mmol/L)	5.7	2.7-7.1
Creatinine (µmol/L)	53	58-94
C-reactive protein (mg/L)	219.1	<1.4

**Figure 1:**
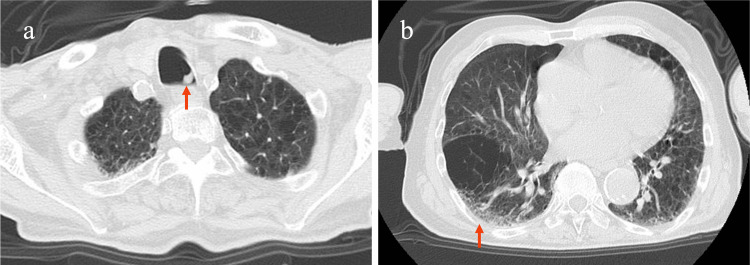
Chest CT of the patient Chest CT shows (a) aspirated sputum in the main bronchus (red arrow) and (b) mild lung infiltration (red arrow) with underlying lung damage caused by COPD. COPD, chronic obstructive pulmonary disease

Chest CT revealed aspirated sputum in the main bronchus (Figure 1a), mild lung infiltration, and old lung damage caused by COPD (Figure 1b).

**Figure 2:**
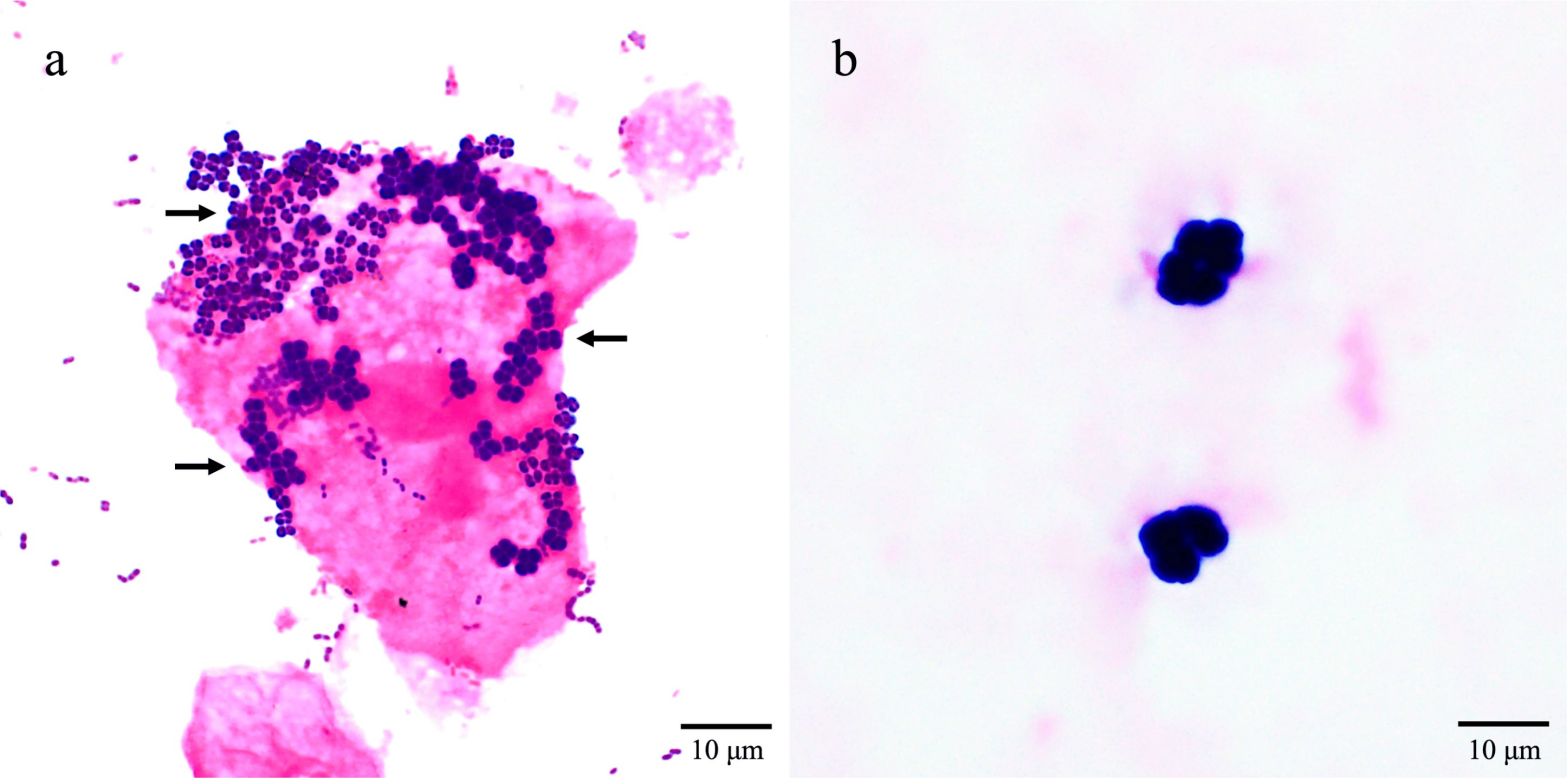
Gram-stained sputum and blood smears showing the appearance of Sarcina ventriculi on microscopy (a) Sputum smear showing large Gram-positive cocci in clusters of tetrads (black arrows; magnification ×1,000). (b) Gram-stained blood smear sample obtained from a positive anaerobic blood culture bottle after 47 hours of culture showing large Gram-positive clusters of tetrads (magnification ×1,000). Due to the large size of the Gram-positive clusters of tetrads, clear focus could not be achieved. The samples were collected immediately prior to initiating antibiotic treatment.

Sputum, urine, and blood samples were submitted for microbiological testing. The Miller and Jones visual classification of the sputum sample was P3, and sputum smears were determined to be class 3 according to the Geckler classification, both indicating purulent sputum. Large Gram-positive cocci clustered in tetrads were present in the sputum (Figure 2a), clearly distinguishable from staphylococci. No significant bacteria were detected on the urine culture. Two sets of blood culture samples were obtained using BD BACTEC Plus Aerobic/F and Anaerobic/F blood culture bottles (Becton, Dickinson and Company, Franklin Lakes, NJ, USA), with approximately 10 mL of blood added to each bottle. The blood culture bottles were promptly transported to the microbiological laboratory and incubated in the BD BACTEC FX blood culture system for five days at 35°C. The anaerobic blood culture bottle showed a positive signal at 47 hours. Gram staining of the blood culture revealed large Gram-positive cocci in clusters of tetrads with difficulty in obtaining a clear focus on microscopy owing to their large size (Figure 2b), morphologically similar to the Gram-positive bacteria that had been seen in the sputum (Figure 2a).

As no potential infectious source other than the respiratory tract was identified, the patient was diagnosed with bacteremia and aspiration pneumonia, and ceftriaxone (CTRX) 2 g daily was initiated. His fever resolved two days after starting CTRX, and his respiratory function recovered to the pre-admission level. He was treated with CTRX for five days and was discharged without further complications.

**Figure 3:**
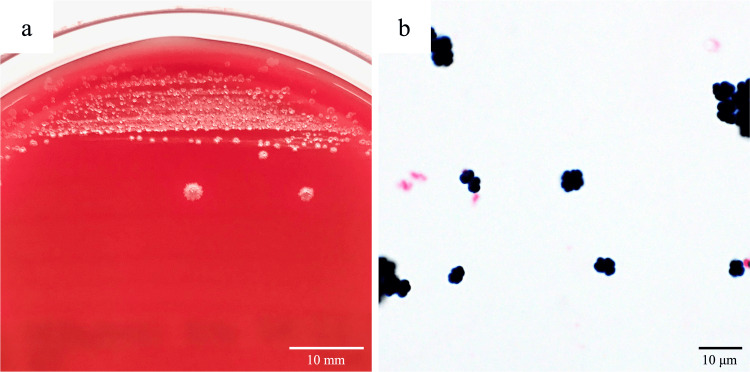
Appearance of Sarcina ventriculi colonies on Brucella HK agar and bacterial morphology on Gram staining (a) Characteristic irregular-shaped, grayish colonies with ragged edges of *S. ventriculi* growing on Brucella HK agar. The colonies were cultured from a blood culture sample plated on Brucella HK agar and incubated at 35°C for 48 hours under anaerobic conditions. (b) Microscopic image of Gram-stained *S. ventriculi* grown on Brucella HK agar (magnification ×1,000).

During bacterial isolation from blood cultures, irregular-shaped, grayish colonies with ragged edges were cultured on Brucella HK agar (RS) (Kyokuto Pharmaceutical Industrial Co., Ltd., Tokyo, Japan) after 48 hours of incubation at 35°C (Figure 3).

Similar colonies were cultured on phenylethyl alcohol-supplemented Brucella HK agar, but not on paromomycin-vancomycin (VCM)-supplemented Brucella HK agar or Bacteroides Bile Esculin agar (Kyokuto Pharmaceutical Industrial Co., Ltd., Tokyo, Japan). Identification using matrix-assisted laser desorption ionization-time of flight mass spectrometry (MALDI-TOF MS) with the MALDI Biotyper Microflex LT/SH (Bruker Daltonics GmbH & Co. KG, Bremen, Germany) and MBT Compass Library 2023 Revision P confirmed *Sarcina *sp. (score value 1.80). Species-level identification was performed using 16S rDNA sequencing by amplifying a 1,447-bp DNA fragment using universal primers 27F and 1492R. The DNA sequence analysis showed 99.65% identity with *S. ventriculi* (DSM 286) 16S rRNA gene (GenBank accession number X76649), and 100% identity with* S. ventriculi *16S ribosomal RNA gene, complete sequence (GenBank accession number AF110272), confirming its identity as *S. ventriculi* [6]. A test for β-lactamase production using BD BBL Cefinase paper discs (Becton, Dickinson and Company) was negative. Antimicrobial susceptibility testing was performed using Brucella broth and Dry Plate Eiken DP1R (Eiken Chemical Co., Ltd., Tokyo, Japan), incubated under anaerobic conditions at 36°C ± 1°C for five days, which revealed minimum inhibitory concentrations (MICs) of ≤0.5 µg/mL for minocycline, levofloxacin, moxifloxacin, and clindamycin (CLDM), and ≤2 µg/mL for metronidazole (MNZ), confirming CLDM and MNZ susceptibility according to the CA-SFM 2019 guidelines [7]. However, the MICs of β-lactams were variable and were classified as indeterminate after five independent tests.

## Discussion

*S. ventriculi* was first described in 1842 after being found in the stomach contents of a patient with vomiting [8]. The individual bacteria are large (1.8-3 μm in diameter) compared with staphylococci (~1 μm in diameter) [3]. The name comes from the Latin “sarcina” meaning “package” or “bundle,” and the bacteria typically clustered in tetrads or octets [9]. The number of bacterial cells per cluster can reach several hundred [10]. Likewise in this case, the Gram-stained smear from the blood culture bottle revealed distinctly large Gram-positive cocci, clearly distinguishable from the stained smear from staphylococci. Furthermore, the characteristic appearance of irregular-shaped, grayish colonies with ragged edges also helps to identify *Sarcina *spp. As a colonizer of the stomach and upper intestinal tract, *S. ventriculi *is well adapted to acidic environments. In low pH (acidic) environments, it exhibits characteristic square or cuboidal cell arrangements, whereas in high pH (alkaline) environments, it exhibits distorted cell shapes and irregular arrangements, and spore formation is induced [11]. The correct nomenclature of this species has been a source of debate. In addition to the two historical species names, *S. ventriculi *and *Zymosarcina ventriculi*, a reclassification based on 16S rRNA phylogeny proposed that the species be transferred to Clostridium ventriculi [10,12]. However, the proposal was ultimately rejected, and the correct name for this bacterial species remains* S. ventriculi *[13]. Other species of the genus *Sarcina *include *Sarcina maxima*, which has also been detected in human feces [14]. Only one *Sarcina *sp. strain is registered in the MALDI-TOF MS library (MBT Compass Library 2023 Revision P). Therefore, 16S rDNA sequencing is required to identify* S. ventriculi* at the species level, as in this study.

No standard method has been established for antimicrobial susceptibility testing of *S. ventriculi*. In a previous report, its antimicrobial susceptibility was determined by the broth dilution method using Brucella broth under anaerobic conditions at 35°C ± 2°C for 48 ± 4 hours according to the CA-SFM 2019 guidelines [7]. Antibiotic susceptible breakpoints in the guidelines were set as follows: penicillin G: 0.25 mg/L; amoxicillin (AMPC): 4 mg/L; AMPC/clavulanic acid: 4/2 mg/L; piperacillin (PIPC): 16 mg/L; PIPC/tazobactam (TAZ): 8/4 mg/L; ticarcillin: 16 mg/L; ticarcillin/clavulanic acid: 8/2 mg/L; ertapenem: 0.5 mg/L; imipenem: 2 mg/L; meropenem: 2 mg/L; VCM: 2 mg/L; CLDM: 4 mg/L; linezolid: 2 mg/L; tigecycline: 4 mg/L; rifampicin: 4 mg/L; MNZ: 4 mg/L; and chloramphenicol: 8 mg/L [7]. In this study, antimicrobial susceptibility testing was also performed according to the CA-SFM 2019 guidelines. Owing to the slow growth of the bacteria, the incubation period was extended to five days. We were unable to determine the MICs of β-lactam antibiotics, which were variable and were classified as indeterminate. Therefore, a modified culture method is required for the determination of the MICs of β-lactam antibiotics. However, MICs for MINO, quinolones, CLDM, and MNZ were consistently low. The isolate was interpreted as susceptible to CLDM and MNZ according to the CA-SFM 2019 guidelines [7]. Considering the patient’s clinical course and previous cases, CTRX could be an effective option for treating *S. ventriculi *bacteremia.

A systematic review reported that *S. ventriculi* has been isolated from the gastrointestinal tract (88%), respiratory tract (5%), urine (4%), and blood (3%) [9]. Various antibiotics and other gastrointestinal agents have been used for the treatment of *S. ventriculi* infections [9]. Regarding the pathogenic roles of *S. ventriculi*, it has been detected in both clinically stable patients with gastrointestinal symptoms and in patients with life-threatening conditions such as emphysematous gastritis or gastric perforation [4]. Common symptoms of *S. ventriculi* infection include abdominal pain, distention, nausea and vomiting, diarrhea, and dyspepsia, but some cases are asymptomatic. Consequently, it is generally treated with gastrointestinal agents, with or without antibiotics, and gastric surgery is rarely required [9]. For the treatment of bacteremia, various antibiotics, including oral AMPC, LVFX, intravenous VCM and PIPC/TAZ, and CTRX (as in the current case), have been used (Table 2) [7,15-17].

**Table 2:** Case reports of Sarcina ventriculi bacteremia ^*^ One set of blood cultures consisted of a pair of aerobic and anaerobic bottles. AMPC, amoxicillin; COPD, chronic obstructive pulmonary disease; CTRX, ceftriaxone; IV, intravenous; LVFX, levofloxacin; ND, not described; PIPC/TAZ, piperacillin/tazobactam; VCM, vancomycin

Reference (year)	Country	Age, sex	Underlying diseases	Suspected infection source	Positive blood culture in submitted blood culture set*	Other samples containing *S. ventriculi*	Antimicrobial therapy	Outcome
Bortolotti et al. [7] (2019)	France	65, M	Cardiac failure, ileocecal resection for acute colonic pseudo-obstruction	Gastrointestinal tract	One anaerobe bottle in one set	ND	IV VCM for five days and IV PIPC/TAZ for 10 days	Survived
Tuuminen et al. [15] (2013)	Finland	48, F	Congenital chloride diarrhea	Gastrointestinal tract	One anaerobe bottle in one set	ND	Oral AMPC for five days	Survived
Elvert et al. (2018) [16] and Elvert et al. [17] (2018)	USA	33, F	Medullary sponge kidney	Gastrointestinal or urogenital tract	One anaerobe bottle in one set	ND	Oral LVFX for 14 days	Survived
This case	Japan	89, M	Past cerebral infarction, atrophic gastritis, COPD	Lower respiratory tract (aspiration pneumonia)	One anaerobe bottle in one set	*Sarcina*-like bacteria in sputum	IV CTRX for five days	Survived

In all previous case reports of bacteremia, the gastrointestinal tract was presumed to be the portal of entry, and the cases had favorable outcomes [7,15-17]. In this case, the patient developed aspiration pneumonia, and the characteristic bacterial morphology was observed in organisms in his sputum; therefore, we presume that *S. ventriculi *entered the patient’s blood via the lungs, which were damaged owing to severe COPD.

## Conclusions

We described a unique case of bacteremia caused by *S. ventriculi* as a complication of aspiration pneumonia in a man with a history of cerebral infarction, atrophic gastritis, and COPD. *S. ventriculi* is a rare cause of bacteremia. A literature review revealed only three previous case reports of *S. ventriculi* bacteremia. In previous case reports, the gastrointestinal tract was the presumed portal of entry into the blood. To our knowledge, *S. ventriculi* bacteremia has not previously been reported as a complication of lower respiratory tract infection. This case suggests that the respiratory tract can serve as a portal of entry for* S. ventriculi* into the blood. Further case studies are required to improve understanding of the pathogenicity and optimal antimicrobial treatment of *S. ventriculi*.
